# Supplementary material for: The New Insight into the Effects of Different Fixing Technology on Flavor and Bioactivities of Orange Dark Tea
Source: Molecules. 2023 Jan 20;28(3):1079. doi: 10.3390/molecules28031079 (PMC9920512; doi:10.3390/molecules28031079)
Supplement: Supplementary file 1 [file molecules-28-01079-s001.zip › molecules-2160126-supplementary.pdf]

Supplementary Materials:

Table S1. Moisture content of tea and peels after different fixing methods (%).

Moisture	Tea	Peel
HA	4.79 ± 0.01	4.58 ± 0.25
ST	4.60 ± 0.01	4.20 ± 0.08
SL	4.29 ± 0.01	5.36 ± 0.31
